# Effect of Digital Health Interventions on College Students’ Lifestyle Behaviors: Systematic Review

**DOI:** 10.2196/82192

**Published:** 2026-02-04

**Authors:** Qingyuan Zhou, Jiajun Jiang, Zhihua Yin, Ruishi Fan

**Affiliations:** 1 College of Physical Education and Health East China Normal University Shanghai China

**Keywords:** digital health interventions, college students, lifestyle behaviors, systematic review, physical activity, sedentary behavior, diet, sleep

## Abstract

**Background:**

College students undergo a critical transition from adolescence to adulthood, during which lifestyle behaviors such as physical activity, sedentary behavior, diet, and sleep are key determinants of long-term health. Digital health interventions (DHIs) are increasingly recognized as a promising strategy for improving these behaviors among college students.

**Objective:**

This systematic review aims to evaluate the effectiveness and applicability of DHIs targeting lifestyle behaviors among college students by analyzing intervention objectives, modalities, functionalities, outcomes, and other key characteristics.

**Methods:**

In accordance with the PRISMA (Preferred Reporting Items for Systematic Reviews and Meta-Analyses) 2020 guidelines, multiple scientific databases, including Scopus, Web of Science, PubMed, MEDLINE, PsycINFO, SPORTDiscus, ProQuest Central, APA PsycArticles, ERIC, and Academic Search Premier, were searched for studies published between January 2010 and December 2025 (initial search: August 5, 2025; updated search: December 27, 2025). The inclusion criteria were original empirical studies on DHIs targeting lifestyle behaviors (physical activity, sedentary behavior, diet, and sleep) among college students, published in English. Studies focusing on nondigital interventions, lacking sufficient methodological details, or not reporting lifestyle behavior–related outcomes were excluded. Quality assessment was conducted in 2 stages: all studies were first evaluated using the Mixed Methods Appraisal Tool (2018 version), followed by Risk of Bias 2 for randomized controlled trials and Joanna Briggs Institute critical appraisal tools for nonrandomized studies. A narrative synthesis was used to present and synthesize the findings.

**Results:**

A total of 2998 records were retrieved, of which 46 publications met the inclusion criteria. These included 30 (65%) studies related to physical activity, 26 (57%) studies to diet, 10 (22%) studies related to sedentary behavior, and 6 (13%) studies related to sleep. This review enabled an examination of the effects of DHIs on college students’ lifestyle behaviors. DHIs primarily used mobile apps, web-based platforms, and mobile communication technologies, with core functionalities such as education, guidance, monitoring, and prompting. DHIs were more effective in improving physical activity and diet; however, evidence for reducing sedentary behavior and improving sleep remained limited. Of the 46 studies, 31 (67%) reported positive effects, with larger sample sizes and intervention durations of 8-16 weeks being associated with more favorable outcomes.

**Conclusions:**

This review focuses on college students, addressing a gap in the literature that often centers on general adult populations. Unlike previous reviews that focus on a single behavior, this study integrates multiple lifestyle behaviors and evaluates DHIs across diverse modalities and functionalities. These contributions help refine future DHIs for college students and inform health promotion strategies in higher education. Although DHIs show potential for improving lifestyle behaviors, evidence of their long-term effectiveness remains limited. Future interventions should prioritize multibehavior integration, interactivity, and population-differentiated design to enhance precision, sustainability, and equity. This study has several limitations, including issues related to sample representativeness, intervention refinement, and methodological rigor.

**Trial Registration:**

PROSPERO CRD420251119078; https://www.crd.york.ac.uk/PROSPERO/view/CRD420251119078

## Introduction

College students are in a critical developmental stage characterized by the transition from adolescence to adulthood, during which they encounter multiple challenges, including increased academic demands and evolving social roles. Evidence suggests that college students often exhibit insufficient self-management capacity related to healthy lifestyle behaviors [1], with inadequate physical activity, prolonged sedentary behavior, irregular diet patterns, and sleep disturbances being particularly prevalent. Previous research has demonstrated that health behaviors established during this developmental period tend to exhibit substantial stability and continuity over time [2]. The adoption of unhealthy lifestyle behaviors during this stage has been shown to significantly increase the risk of chronic diseases, depression, and anxiety later in adulthood [3,4]. Therefore, the implementation of early and effective interventions targeting these 4 key lifestyle behaviors among college students is of substantial public health significance [1].

With the rapid advancement of digital technologies and the widespread adoption of smart devices, digital health interventions (DHIs) have emerged as an innovative approach to health promotion and are increasingly recognized as an important means of improving lifestyle behaviors among college students [5,6]. Particularly in the post–COVID-19 era, DHIs have demonstrated greater adaptability and broader application potential than traditional face-to-face health intervention models [7-9]. In recent years, a growing body of empirical evidence has shown that DHIs are effective in promoting physical activity among college students [10-12], reducing sedentary time [13,14], improving diet behaviors [15,16], and enhancing sleep quality [17,18]. These interventions—encompassing mobile apps, wearable devices, online platforms, and social media—offer several advantages, including low cost, high scalability, and a high degree of personalization [19,20], and have been shown to enhance user engagement and facilitate sustained behavior change [21,22]. Concurrently, advancements in emerging technologies, such as artificial intelligence, continue to drive the refinement of DHI implementation strategies and further enhance intervention effectiveness [23].

However, the existing body of research on DHIs targeting lifestyle behaviors among college students remains subject to several limitations. On the one hand, the majority of original intervention studies have focused on single lifestyle behaviors or specific technological modalities, with a relative lack of comprehensive designs that integrate multiple behaviors and intervention approaches. At the same time, key intervention dimensions—such as functional characteristics, intervention duration, participant demographics, and adherence—have yet to reach unified standards or methodological consensus [24,25]. On the other hand, existing systematic reviews and meta-analyses in this field also demonstrate limitations in terms of specificity and methodological rigor. First, systematic syntheses that specifically target the college student population remain relatively scarce, with insufficient attention paid to lifestyle behaviors such as sedentary behavior and sleep. Second, existing analyses have not adequately synthesized the combined effects of multiple lifestyle behaviors across diverse DHI intervention formats [26].

In light of the current research context and identified limitations, this review is guided by the following research questions: (1) What is the current state of the literature on DHIs targeting 4 key lifestyle behaviors among college students (physical activity, sedentary behavior, diet, and sleep)? (2) What are the specific implementation strategies and modalities of DHIs addressing these behaviors? (3) To what extent are DHIs effective in influencing these 4 target lifestyle behaviors among college students? Through a comprehensive synthesis and analysis of relevant primary research evidence, this review will explicitly consider the characteristics of college students as “digital natives” [27]. The review will systematically examine the forms, functions, and key components of different DHIs, and comprehensively evaluate their effects on the 4 target behaviors that are closely related to college student health. This review aims to clarify the applicability and effectiveness of DHIs within this population, thereby providing evidence-based recommendations for optimizing DHI tools, informing health promotion strategies in higher education settings, and guiding future research.

## Methods

### Search Strategy

This systematic review was prospectively registered in PROSPERO (International Prospective Register of Systematic Reviews) on August 4, 2025 (registration number: CRD420251119078), and the reporting of the review findings adheres to the PRISMA (Preferred Reporting Items for Systematic Reviews and Meta-Analyses) 2020 guidelines (see Multimedia Appendix 1). A comprehensive literature search was conducted across 10 major English-language electronic databases, including Scopus, Web of Science, PubMed, ProQuest Central, and 6 databases accessed via the EBSCOhost platform (MEDLINE, PsycINFO, SPORTDiscus, APA PsycArticles, ERIC, and Academic Search Premier), with Google Scholar used as a supplementary search source. In addition, the reference lists of relevant articles were screened to identify potentially missed studies. The initial search was completed on August 5, 2025, covering studies published between January 1, 2010, and June 1, 2025, for primary study identification, and an updated search was conducted on December 27, 2025, to capture studies published within the most recent 6 months; the same search strategy was applied consistently across both searches. No published search filters were used, and the search strategy was neither adapted from nor reused, in whole or in part, from previous reviews. The search strategy was initially developed by the authors and subsequently peer reviewed by an experienced searcher with expertise in scientific information retrieval. Beyond these approaches, no study registries were searched, no purposeful searching or browsing (eg, table of contents screening, print conference proceedings, or website searches) was conducted, and no additional information was sought by contacting authors, experts, manufacturers, or other relevant parties.

The literature search strategy was systematically developed in accordance with the PRISMA-S (Preferred Reporting Items for Systematic Reviews and Meta-Analyses—Search Extension) guidelines to ensure transparency and reproducibility of the search process. The strategy combined Medical Subject Headings terms with free-text terms and constructed keyword combinations around 3 core concepts: (1) intervention formats (eg, digital health, digital intervention, eHealth, and mobile health [mHealth]); (2) target behaviors (eg, physical activity, sedentary behavior, sleep, and diet); and (3) study populations (eg, university students, college students, and undergraduate students). Boolean operators (AND and OR) were applied to balance search sensitivity and specificity. Using Scopus as an example, the search query was as follows: TITLE-ABS-KEY (“digital health” OR “eHealth” OR “mHealth” OR “mobile health” OR “digital intervention” OR “health app”) AND TITLE-ABS-KEY (“college students” OR “university students” OR “undergraduate students” OR “young adults”) AND TITLE-ABS-KEY (“lifestyle behavior” OR “health behavior” OR “physical activity” OR “exercise” OR “diet” OR “nutrition” OR “sleep” OR “sedentary behavior”). The complete English-language search terms used across all databases are provided in Multimedia Appendix 2. To ensure comprehensive coverage, no geographical restrictions were applied during the literature search, allowing for the inclusion of relevant studies from diverse global regions.

### Inclusion and Exclusion Criteria

The study inclusion criteria were developed in accordance with the PICOS (Population-Intervention-Comparison-Outcome -Study Design) framework, as outlined in Textbox 1.

The exclusion criteria were as follows: (1) The study population was not explicitly identified as “college students,” “university students,” or individuals enrolled in higher education institutions. (2) The nondigital components constituted the majority of the intervention (≥50%), or the study relied solely on wearable devices for passive behavioral monitoring without incorporating feedback mechanisms or active intervention strategies. (3) The study did not implement a behavioral intervention, or the intervention description lacked sufficient detail to determine its content and implementation procedures. (4) Studies that did not report any lifestyle behavior–related outcome measures. (5) Conference abstracts, theses, unpublished manuscripts, and other forms of gray literature. (6) Full-text articles were unavailable, or the publication was not in English.

**Study inclusion criteria.**

**1. Population**

- Participants were required to be aged ≥18 years and explicitly identified as “college students,” “university students,” or “young adults enrolled in higher education.”

**2. Intervention**

- Studies were required to evaluate at least one health intervention primarily delivered through digital health technologies and targeting lifestyle-related behaviors. Digital health interventions included, but were not limited to, mobile apps, web-based platforms, SMS text message reminders, online courses, virtual coaches, digital gamification strategies, social media, and other eHealth/mHealth tools.

**3. Comparison**

- The presence of a control group was not mandatory; all original studies reporting intervention effects were eligible for inclusion.

**4. Outcomes**

- The primary outcomes included lifestyle behavior indicators, specifically physical activity, sedentary behavior, diet, and sleep.
- Secondary outcomes included physical and mental health indicators, such as weight, waist circumference, and self-efficacy.

**5. Study design**

- Original empirical studies targeting 1 or more of the 4 lifestyle behavior domains among college students and implementing digital health interventions were included.
- No restrictions were placed on study design; however, intervention content, participant characteristics, and relevant outcome measures were required to be clearly reported.

### Study Selection

All retrieved records were imported into EndNote 20 (Clarivate Plc) reference management software for duplicate removal and standardized record numbering. Subsequently, 2 reviewers (QYZ and JJJ) independently screened titles and abstracts for initial eligibility. Records that passed the initial screening were subjected to full-text assessment to determine final eligibility for inclusion. To ensure standardization and consistency in the screening process, all reviewers received standardized training on the predefined inclusion and exclusion criteria. Interrater reliability between the 2 reviewers was assessed using the Cohen κ coefficient, yielding a value of 0.86, which indicates a high level of screening agreement. In cases of disagreement regarding individual records, a third reviewer (ZHY) was consulted to facilitate discussion and achieve a final consensus.

### Data Extraction and Synthesis

To ensure standardization and consistency in the data extraction process, the research team developed a structured data extraction form in advance, covering the study title, first author, publication year, study region, study design, intervention population characteristics, intervention protocol characteristics, outcome measures, intervention effectiveness, and study conclusions. The data extraction form was pilot-tested using 5 studies to assess its feasibility. During the formal data extraction process, 2 reviewers (QYZ and JJJ) independently extracted the data. In cases of missing data or discrepancies in interpretation, a third reviewer (ZHY) was consulted to resolve disagreements. The final extracted data were consolidated into a standardized table and are presented in Multimedia Appendix 3.

### Quality Assessment

All included studies were initially assessed for methodological quality using the Mixed Methods Appraisal Tool (MMAT, 2018 version) to obtain an overall preliminary appraisal of study quality. The MMAT is designed to evaluate 5 categories of study designs: qualitative research (QR), quantitative randomized controlled trials (QRCTs), quantitative nonrandomized studies (QNRSs), quantitative descriptive studies (QDSs), and mixed methods studies (MMSs), each comprising 5 appraisal criteria [28]. To enhance specificity and methodological rigor, the Risk of Bias 2 (RoB 2) tool was further applied to assess the risk of bias in QRCTs. For all other study designs, the Joanna Briggs Institute (JBI) critical appraisal tools were applied. This 2-stage quality assessment approach was intended to balance breadth and depth in methodological evaluation. Quality assessments were conducted independently by 2 reviewers (QYZ and JJJ), with discrepancies resolved through discussion. To ensure consistency, both reviewers received standardized training on the MMAT, RoB 2, and JBI critical appraisal tools and completed pilot scoring exercises before the formal assessment.

## Results

### Screening and Inclusion Results

#### Search and Screening Results

**Figure 1:**
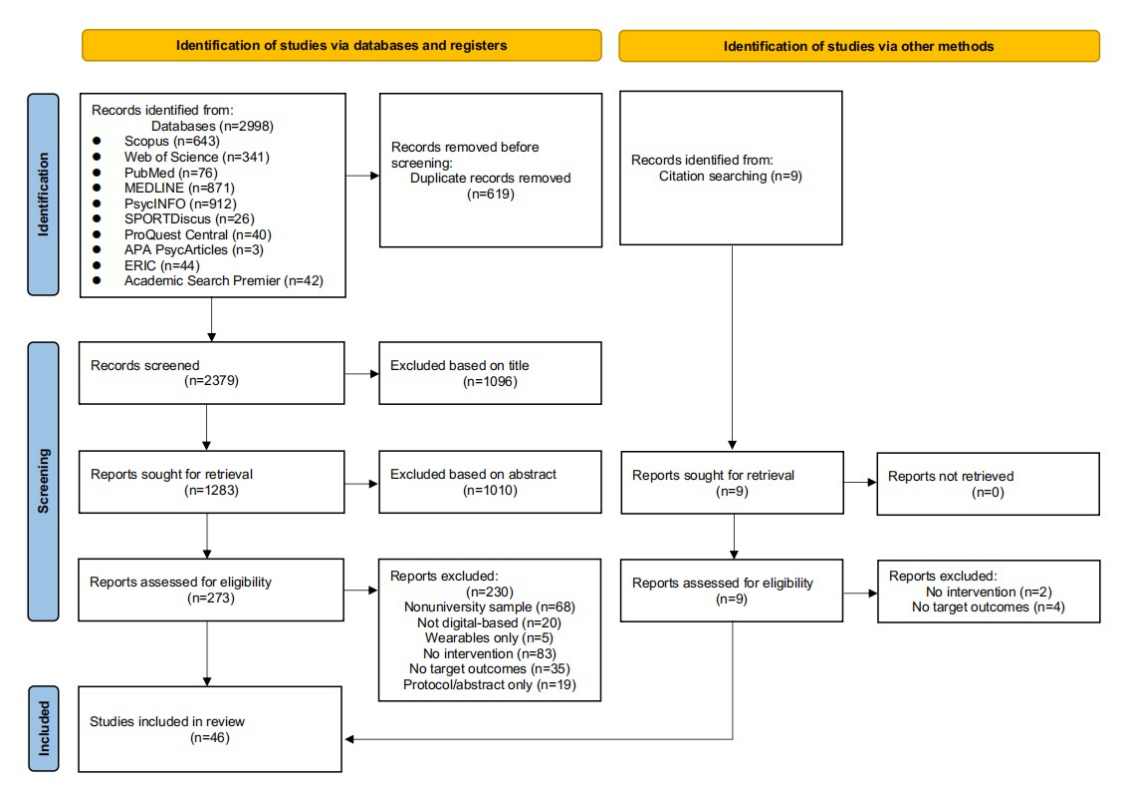
PRISMA flowchart of the study selection process.

In this study, a total of 2998 records were retrieved from 10 major English-language databases. After deduplication and initial title and abstract screening, 273 articles were selected for full-text review. Based on the predefined exclusion criteria, exclusions were made for the following reasons: nonuniversity samples (n=68); interventions not primarily digital-based (n=20); wearable devices only, without active intervention components (n=5); absence of behavioral interventions (n=83); lack of relevant behavioral outcomes (n=35); and protocol or abstract only (n=19). Additionally, 3 more articles were identified through manual reference tracing of relevant review papers. Ultimately, 46 publications met the inclusion criteria and were included in the final analysis, as depicted in Figure 1.

#### Quality Assessment Results

**Figure 2:**
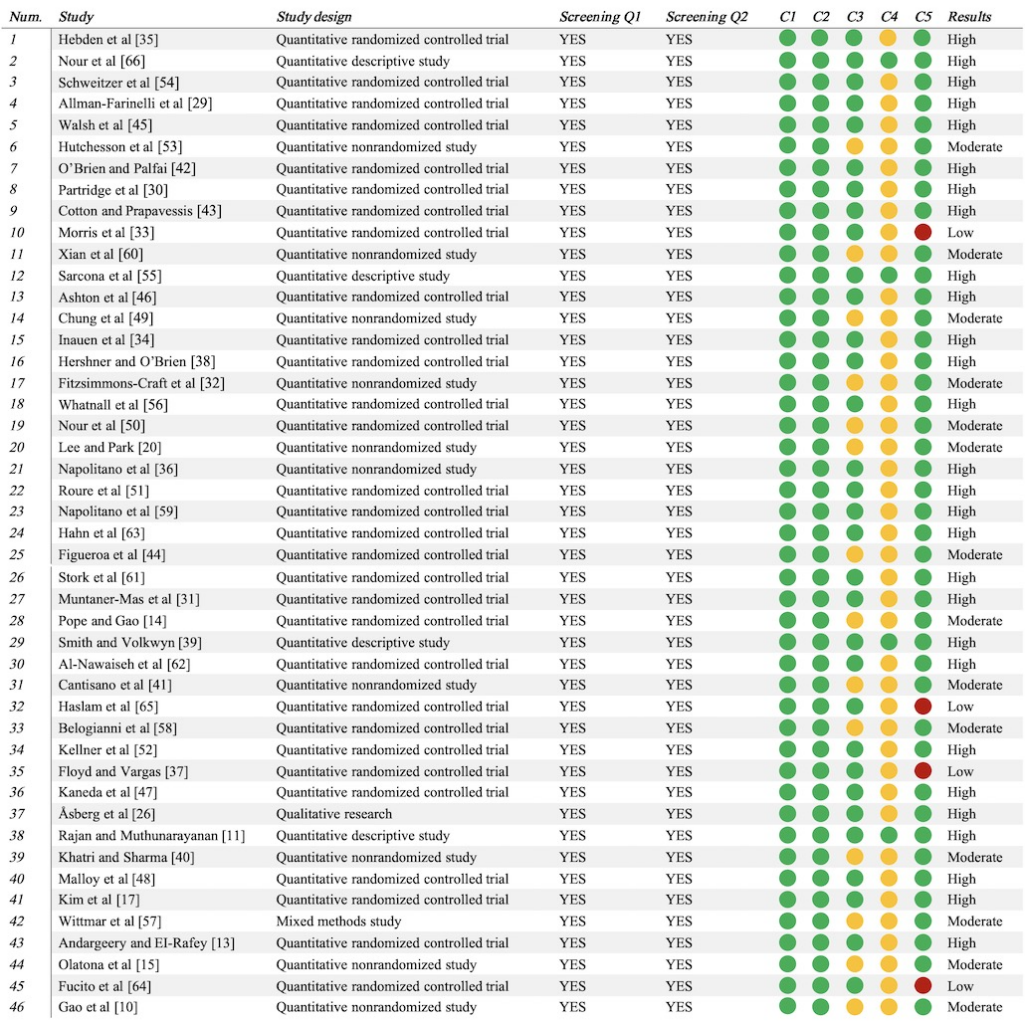
Quality assessment results of the Mixed Methods Appraisal Tool.

**Figure 3:**
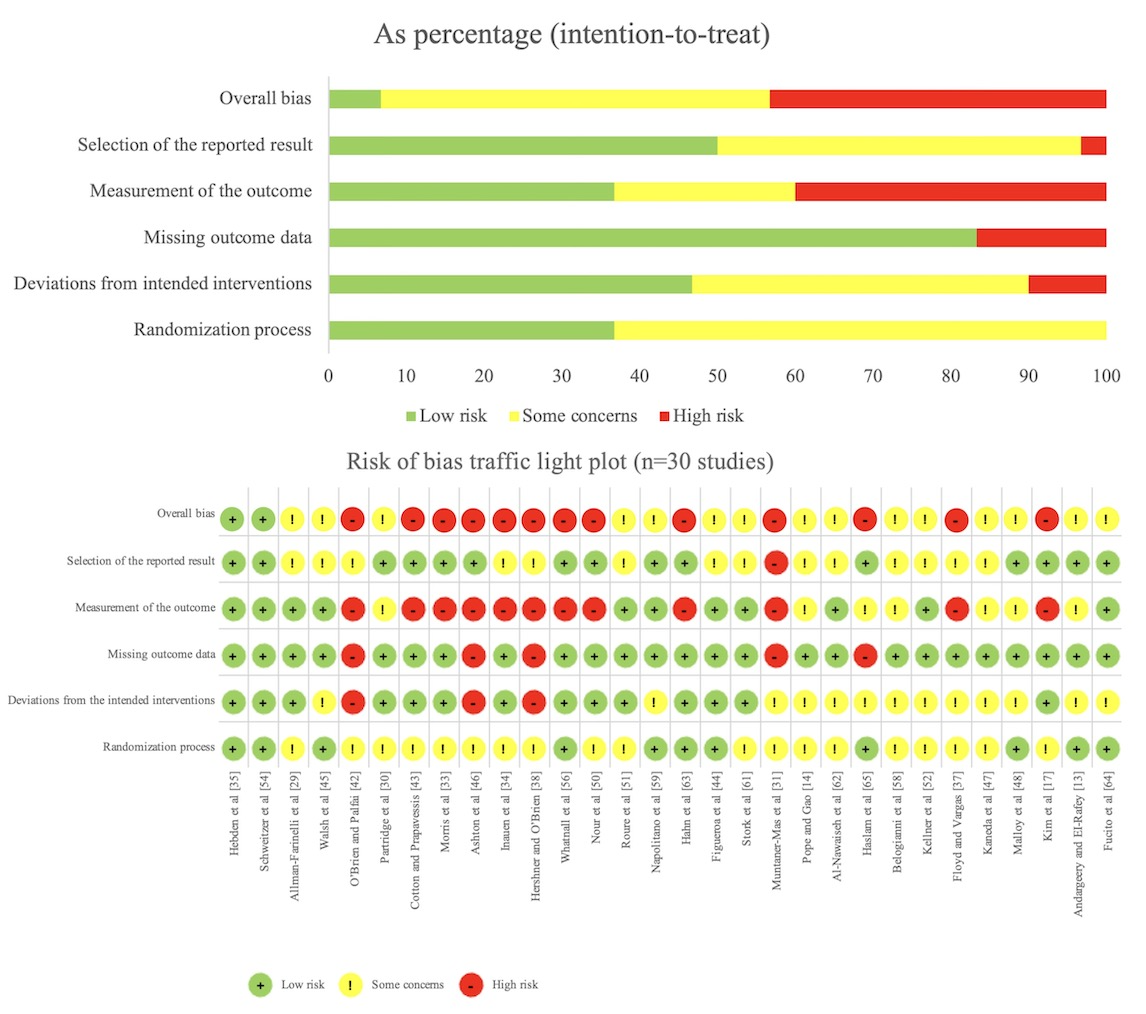
Quality assessment results of the Risk of Bias 2 tool.

**Figure 4:**
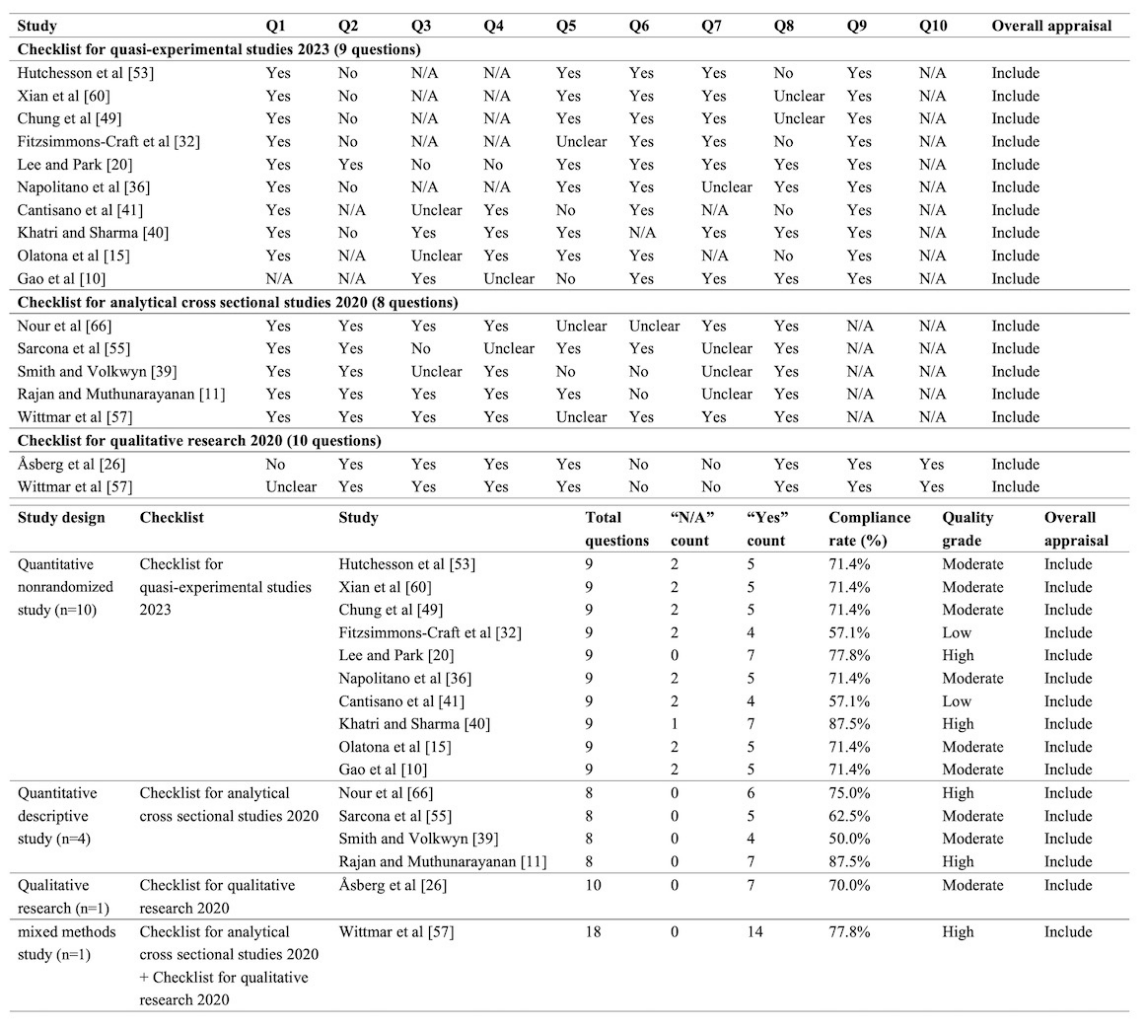
Quality assessment results of Joanna Briggs Institute critical appraisal tools. N/A: not applicable.

Following 2 rounds of quality assessment, the first-round MMAT evaluation indicated that the 46 included studies demonstrated an overall high level of methodological quality. Specifically, 28 (61%) studies were rated as high quality, 14 (30%) as moderate quality, and 4 (9%) as low quality (see Figure 2; also see [10,11,13-15,17,20,26,29-66]). Major methodological concerns identified during the assessment were primarily concentrated in MMAT items C4 and C5. Item C4 was primarily related to the implementation of blinding procedures, the adequacy of outcome interpretation, and the control of risk of bias, whereas item C5 reflected issues such as insufficient intervention adherence and the lack of rigorous statistical analyses. In the second round of assessment, the RoB 2 tool was applied to evaluate 30 QRCTs, indicating that the primary sources of bias were related to outcome measurement, deviations from intended interventions, and the handling of missing outcome data (see Figure 3; see also [13,14,17,29-31,33-35,37,38,42-48,50-52,54,56,58,59,61-65]). Concurrently, the JBI critical appraisal of the remaining 16 studies indicated that key factors influencing study quality primarily included sample representativeness, intervention adherence, and the objectivity of outcome measurement (see Figure 4; see also [10,11,15,20,26,32,36, 39-41,49,53,55,57,60,66]).

The emergence of these methodological issues can be primarily attributed to 2 factors. On the one hand, the behavioral nature of DHIs makes the implementation of blinding inherently challenging, and several key behavioral outcomes rely on participant self-report measures. On the other hand, relatively high dropout rates associated with DHIs contribute to issues such as low intervention adherence and elevated loss-to-follow-up rates in some studies. When combined with insufficiently rigorous statistical analyses, these challenges may result in suboptimal handling of missing data or deviations from intended interventions. Although studies rated as moderate to low quality constitute a notable proportion of the included literature, it is important to recognize that many of their methodological limitations are closely related to the inherent characteristics of DHIs. Moreover, many of these studies primarily aimed to explore the feasibility and applicability of DHIs rather than to provide definitive evidence of intervention efficacy. Therefore, these studies retain substantial value for informing future research and intervention development. Given these considerations, no studies were excluded from this review solely based on methodological quality. Instead, all eligible studies were included, and findings from risk-of-bias assessments were systematically incorporated into the narrative synthesis. This approach allows for a comprehensive presentation of the current evidence landscape while explicitly identifying both its strengths and limitations.

#### Data Extraction Results

This review included a total of 46 studies. The basic characteristics of the included studies are summarized in Table 1, with detailed data extraction results provided in Multimedia Appendix 3. Given the substantial heterogeneity among the included studies with respect to study design, target behaviors, intervention formats, core functions, and primary outcome measures, as well as variations in methodological quality, a meta-analysis was not conducted. Instead, a comprehensive analysis was performed using descriptive synthesis and comparative approaches. By systematically organizing and describing key characteristics of DHIs—including intervention targets, participant characteristics, sample sizes, formats, functions, durations, outcomes, and effects—this review delineates the overall patterns and heterogeneity within the field. Specific details are elaborated in the subsequent sections and illustrated through relevant tables, charts, and figures.

**Table 1:** Summary of data extraction from included studies.

Study design and relevant studies		Total completed, N	Participant age (years), mean (SD)	Intervention(s)	Target behavior(s)	Function	Effectiveness		
**Quantitative randomized controlled trial**									
	Hebden et al [35]	46	22.8 (4.6)	SMS text messages, emails, smartphone apps, and internet forums	Physical activity Sedentary behavior Diet	Prompting Education Guidance	Limited^a^		
	Schweitzer et al [54]	106	19.7 (0.73)	Email	Physical activity Diet	Guidance Education Prompting	Yes^b^		
	Allman-Farinelli et al [29]	202	27.7 (4.9)	Coaching calls, SMS text messages, emails, apps, and downloadable website resources	Physical activity Diet	Guidance Prompting Education	Yes		
	Walsh et al [45]	55	20.55 (2.07)	Smartphone app	Physical activity	Monitoring Feedback	Yes		
	O’Brien and Palfai [42]	148	19.24 (1.16)	Web and SMS text messages	Diet	Education Prompting Guidance	Limited		
	Partridge et al [30]	248	27.0 (4.0)	Coaching calls, SMS text messages, emails, smartphone apps, and website	Physical activity Diet	Education Guidance	Yes		
	Cotten and Prapavessis [43]	56	21.19 (4.19)	SMS text messages	Sedentary behavior	Prompting Guidance	Limited		
	Morris et al [33]	112	20.5 (1.95)	Internet	Sleep	Education Guidance	Yes		
	Ashton et al [46]	47	22.1 (2.0)	Website, wearable device, and Facebook support group	Physical activity Diet	Guidance Education Interaction	Limited		
	Inauen et al [34]	141	27.5 (8.6)	App	Diet	Interaction Monitoring	Limited		
	Hershner and O’Brien [38]	358	21.9 (4.1)	Website	Sleep	Education	Yes		
	Whatnall et al [56]	90	22.4 (4.0)	Website	Diet	Education Guidance	Limited		
	Nour et al [50]	47	24.8 (3.4)	Self-monitoring app, gamified app, and social media (Facebook)	Diet	Monitoring Interaction	Limited		
	Roure et al [51]	60	20.8 (1.3)	Exergame	Physical activity	Immersion Engagement	Yes		
	Napolitano et al [59]	283	23.3 (4.4)	Facebook and SMS text messages	Physical activity Diet	Prompting Feedback	Yes		
	Hahn et al [63]	192	20.2 (2.4)	App	Physical activity Diet	Monitoring	No^c^		
	Figueroa et al [44]	93	20.2 (2.47)	App and SMS text messages	Physical activity	Prompting Feedback Monitoring	Yes		
	Stork et al [61]	46	24.0 (5.0)	App	Physical activity	Guidance Monitoring	Yes		
	Muntaner-Mas et al [31]	66	23.1 (4.0)	App	Physical activity	Guidance	Yes		
	Pope and Gao [14]	42	21.6 (NR)^d^	App and Facebook	Physical activity Sedentary behavior	Monitoring Education Prompting	Yes		
	Al-Nawaiseh et al [62]	114	21.1 (2.2)	App	Physical activity	Monitoring Feedback	Yes		
	Haslam et al [65]	141	21.7 (2.0)	Website	Diet	Feedback Education	No		
	Belogianni et al [58]	65	23.01 (3.82)	Website	Physical activity Sedentary behavior Diet	Education Immersion	No		
	Kellner et al [52]	34	22.31 (2.59)	SMS text messages	Sedentary behavior	Prompting	Yes		
	Floyd and Vargas [37]	55	19.9 (0.97)	App	Sleep	Guidance Education	Yes		
	Kaneda et al [47]	46	20.8 (1.2)	E-learning and exercise video	Physical activity Sedentary behavior	Education Guidance	No		
	Malloy et al [48]	46	21.34 (2.02)	Social media	Physical activity Diet	Education Prompting Guidance	Limited		
	Kim et al [17]	60	21.9 (1.43)	Virtual reality	Sleep	Immersion Guidance	Yes		
	Andargeery and El-Rafey [13]	220	19.97 (2.61)	Mobile health tools and videos	Physical activity Diet Sleep	Education Guidance Monitoring	Yes		
	Fucito et al [64]	98	21.16 (1.75)	Wearable devices, website, and smartphone	Sleep	Monitoring Guidance Feedback	Yes		
**Quantitative nonrandomized study**									
	Hutchesson et al [53]	12	22.8 (3.2)	Website, emails, online forum, smartphone app, and SMS text messages	Physical activity Sedentary behavior Diet	Feedback Education Interaction	Yes		
	Xian et al [60]	167	25.0 (4.0)	Reality game	Physical activity	Immersion Prompting	Yes		
	Chung et al [49]	12	19.8 (1.0)	Fitbit, Twitter, and gamification	Physical activity Diet	Monitoring Interaction Prompting	Yes		
	Fitzsimmons-Craft et al [32]	2454	22.89 (6.59)	Online platforms	Diet	Screening Guidance	Yes		
	Lee and Park [20]	59	22.0 (2.0)	Apps and wearable devices	Physical activity Diet	Monitoring Guidance	Yes		
	Napolitano et al [36]	20	18.3 (0.72)	Digital learning modules	Physical activity Sedentary behavior Diet	Monitoring Feedback	Limited		
	Cantisano et al [41]	16	20.69 (1.74)	eHealth tools	Physical activity Diet	Education	Limited		
	Khatri and Sharma [40]	500	20.74 (1.77)	App	Sedentary behavior	Monitoring Feedback Guidance	Yes		
	Olatona et al [15]	1182	Unclear	Social media	Diet	Education Guidance	Yes		
	Gao et al [10]	456	21.5 (1.4)	Artificial intelligence–powered gamification	Physical activity	Interaction	Yes		
**Quantitative descriptive study**									
	Nour et al [66]	401	27.7 (4.9)	Telephone, website, smartphone app, and SMS text messages	Diet	Guidance Education	Yes		
	Sarcona et al [55]	230	22.0 (3.0)	Mobile health apps	Physical activity Diet	Monitoring	Yes		
	Smith and Volkwyn [39]	192	22.7 (3.7)	App	Physical activity	Monitoring Feedback	Yes		
	Rajan and Muthunarayanan [11]	680	23.82 (1.62)	App	Physical activity Diet	Monitoring Education Screening	Yes		
**Qualitative research**									
	Åsberg et al [26]	50	31.3 (6.4)	SMS text messages	Physical activity Sedentary behavior Diet	Guidance Education Feedback	Limited		
**Mixed methods study**									
	Wittmar et al [57]	142	24.0 (4.0)	Web application	Physical activity	Education Interaction	Yes		

^a^Limited: limited evidence of effectiveness, based on reported effect measures, CIs, and authors’ conclusions (see Multimedia Appendix 3).

^b^Yes: evidence of effectiveness, based on reported effect measures, CIs, and authors’ conclusions (see Multimedia Appendix 3).

^c^No: no evidence of effectiveness, based on reported effect measures and authors’ conclusions (see Multimedia Appendix 3).

^d^NR: not reported.

**Figure 5:**
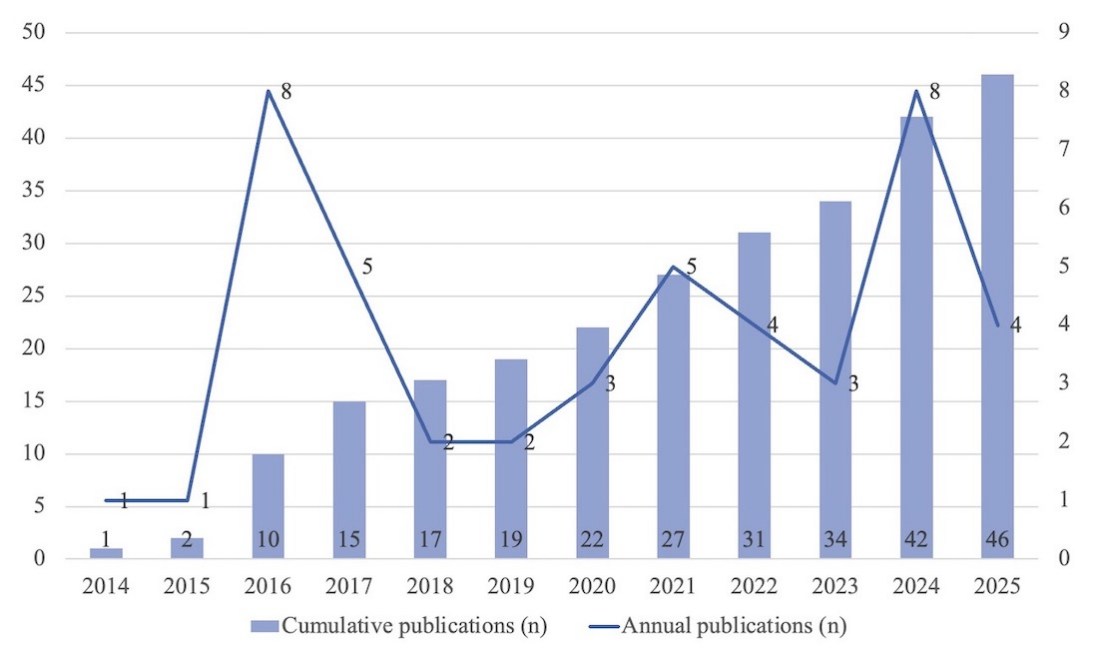
Annual and cumulative publication counts of the included studies.

In terms of annual distribution (see Figure 5), the number of studies during the early period (2014-2015) was low, with only 1 publication per year. Since 2016, the number of publications increased markedly, reaching a first minor peak in 2016 (n=8), possibly associated with the rapid adoption of smartphones and mobile apps among college students. From 2017 to 2020, the number of studies fluctuated between 1 and 5 annually, maintaining an overall moderate level. The number increased again and stabilized in 2021-2022, declined slightly in 2023, reached a second peak in 2024 (n=8), and remained high in 2025 (n=4). Publications from the last 5 years accounted for more than half of all studies identified.

The regional and country distribution of the included studies demonstrates a clear geographical concentration. At the regional level, most studies were conducted in North America (n=18, 39%), followed by Oceania (n=10, 22%) and Europe (n=9, 20%). Asia accounted for 6 (13%) studies, while Africa contributed the smallest share with 3 (7%) studies. At the country level, the United States recorded the highest number of publications (n=15, 33%), followed by Australia (n=9, 20%). The United Kingdom, Germany, Canada, South Korea, and India each contributed 2 studies. The remaining countries were represented by a single study, indicating a relatively dispersed distribution beyond the leading contributors.

The distribution of study design types among the included studies exhibited a clear structural pattern. The largest proportion comprised QRCTs (n=30, 65%). This was followed by QNRSs (n=10) and QDSs (n=4), which were primarily used for exploratory analyses and descriptive accounts of phenomena. By contrast, QR and MMS were represented by only 1 article each, accounting for less than 2% of the total. Overall, DHI studies addressing college students’ lifestyle behaviors are predominantly quantitative, with a marked preference for QRCTs.

With respect to ethical compliance, all included studies adhered to relevant ethical guidelines, with all 46 (100%) explicitly reporting informed consent procedures and ethics committee approval or review status. Regarding privacy protection and data security, 24 (52%) studies explicitly reported the implementation of protective measures, including secure server storage compliant with data safety standards, encrypted data transmission, data deidentification, and strict access control mechanisms. With respect to adverse events and intervention-related risks, no serious adverse events were reported across the included studies. Only a small number of studies reported minor negative issues related to technology use, such as fluctuations in intervention engagement, higher dropout rates, or reduced compliance attributable to participants’ competing academic or personal commitments. No health risks were identified that were directly attributable to the DHIs.

### Intervention Design and Implementation Results

#### Intervention Objectives

Among the intervention objectives examined in the included studies, 30 addressed physical activity, 26 addressed diet, 10 targeted sedentary behavior, and 6 targeted sleep. Single-behavior interventions accounted for a large proportion of the studies; however, multibehavior crossover interventions were also substantial, with combined physical activity and diet interventions being the most common (n=18). Notably, physical activity was both the most frequent single-behavior intervention target and the primary entry point for multibehavior combined interventions, whereas sleep was relatively underemphasized in intervention design.

#### Intervention Participants

Based on the PROGRESS-Plus (Place of Residence, Race/Ethnicity, Occupation, Gender/Sex, Religion, Education, Socioeconomic Status, Plus Other Relevant Factors) framework, a synthesis of sociodemographic characteristics from 46 DHI studies identified 10 primary participant categories (see Multimedia Appendix 3), including health status (n=46), age (n=45), gender/sex (n=45), education (n=41), occupation (n=39), place of residence (n=36), race/ethnicity (n=28), socioeconomic status (n=14), social capital (n=8), and religion (n=1). The analysis revealed the following: (1) All participants were college students, predominantly aged 18-30 years, which is consistent with typical college student demographics and showed no substantial deviation across studies. (2) Most interventions targeted students with generally healthy status, whereas 14 out of 46 (30%) focused on subpopulations with specific health risks or special needs, such as overweight or obesity, sleep disorders, psychological stress, or disordered eating behaviors. (3) Gender/sex distribution was relatively balanced across studies, whereas education and occupation exhibited limited variability owing to the homogeneity of the study population. (4) By contrast, PROGRESS-Plus dimensions such as race/ethnicity, socioeconomic status, social capital, and religion received notably limited attention, with a lack of systematic analysis from a health equity perspective.

#### Intervention Sample

**Figure 6:**
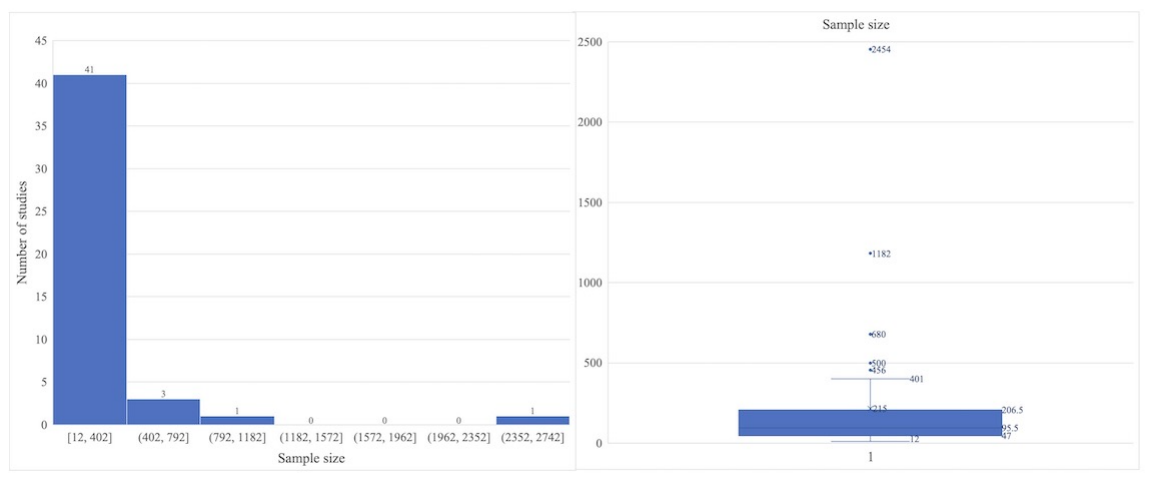
Sample size distribution of the included studies.

The sample sizes of the included studies varied considerably. Histograms indicated that most studies had sample sizes concentrated below 200 participants, with a median of approximately 95, whereas a few studies had small (<50) or extremely large (>400) samples. As shown in Figure 6, box-and-whisker plots further revealed an uneven distribution with long-tailed characteristics. Variations in sample size were closely associated with study design. Rigorous QRCTs typically require larger samples to ensure statistical power and therefore tend to employ medium- to large-scale sample sizes. By contrast, QDSs and QR are more inclined toward small-sample explorations, sometimes recruiting only a few dozen participants, and are more susceptible to selection bias.

#### Intervention Modalities

The intervention formats in the included studies fell into 3 main categories. The first category, *single*, referred to interventions employing only 1 digital health technology (n=29), such as mobile apps. The second category, *multiple*, involved combining multiple digital health technologies within the same intervention (n=10). For example, the TXT2BFiT program integrated phone calls, websites, apps, and SMS text messaging simultaneously to achieve intervention goals. The third category, *combined* (n=7), compared the effectiveness of different combinations of digital health technologies, such as a “web-based nutrition intervention only” versus a “web-based intervention combined with daily SMS text message reminders.” Regarding the types of intervention technologies, these could be categorized into 7 groups: (1) mobile apps, used 21 times; (2) web-based platforms, including websites (13 times), online forums (3 times), and digital learning or eHealth tools (4 times); (3) mobile communications, including SMS text messages (11 times), emails (5 times), and phone calls (3 times); (4) social media (7 times); (5) wearable devices (4 times); (6) gamification and multimedia, including gamification and exergames (5 times), videos (2 times), and virtual reality (1 time); and (7) intelligent technologies, represented only by artificial intelligence (1 time). Overall, mobile apps and web-based platforms were the most frequently used technologies.

#### Intervention Functionalities

The technological functions of the DHIs included in this review exhibited distinct patterns of emphasis. Educational and guidance-related functions predominated across most interventions, followed by monitoring and prompting functions; by contrast, feedback and interactive functions were used less frequently, while immersive, screening, and engagement-related functions were rarely incorporated. Coding these interventions using the Behavior Change Technique Taxonomy version 1 (BCTTv1) indicated that the most frequently employed techniques were “4.1 Instruction on how to perform the behavior” and “5.1 Information about health consequences,” suggesting that current DHIs primarily emphasize foundational behavioral support functions. Further frequency analysis of BCT coding among effective intervention studies (see Table 2) showed that BCTTv1 codes 4.1 (16/87, 18%), 5.1 (14/87, 16%), and 2.3 (13/87, 15%) constituted the core set of techniques, collectively accounting for nearly half of all techniques used in effective interventions.

**Table 2:** Frequency distribution of codes in effective intervention studies (N=87).

Behavior Change Technique Taxonomy version 1 code	Description	Frequency, n (%)
4.1	Instruction on how to perform behavior	16 (18)
5.1	Information about health consequences	14 (16)
2.3	Self-monitoring of behavior	13 (15)
2.2	Feedback on behavior	8 (9)
7.1	Prompts/cues	8 (9)
6.1	Demonstration of behavior	4 (5)
2.1	Monitoring by others (no feedback)	3 (3)
3.1	Social support (unspecified)	3 (3)
5.3	Social/environmental consequences	3 (3)
6.2	Social comparison	3 (3)
12.1	Restructuring physical environment	3 (3)
1.2	Problem solving	2 (2)
1.1	Goal setting (behavior)	1 (1)
1.6	Discrepancy between current behavior and goal	1 (1)
2.4	Self-monitoring of outcomes	1 (1)
2.6	Biofeedback	1 (1)
2.7	Feedback on outcomes	1 (1)
5.6	Emotional consequences	1 (1)
9.1	Credible source	1 (1)

#### Intervention Duration

**Figure 7:**
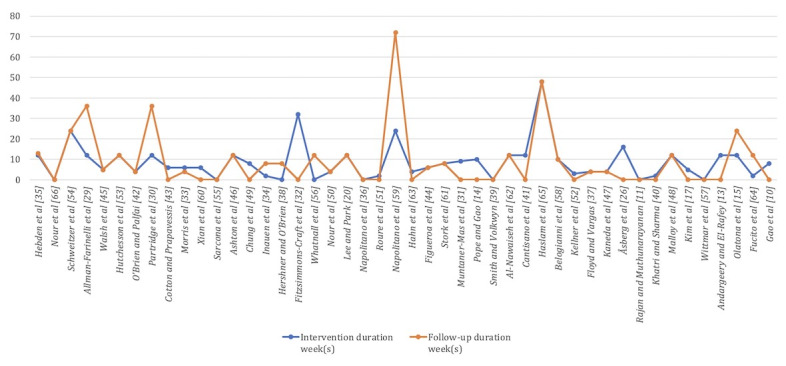
Chart of intervention duration and follow-up duration.

The duration of interventions varied considerably across the included studies (see Figure 7; see also [10,11,13-15,17,20,26,29-66]), with the majority concentrated in the short- to medium-term range (1-16 weeks). Studies involving long-term interventions (>16 weeks) were relatively scarce, with only 4 studies identified. Among these studies, most incorporated follow-up periods, and medium- to long-term interventions were typically associated with more systematic follow-up protocols. With respect to study design, randomized controlled trials predominantly employed interventions of medium duration (8-16 weeks). Among the QDSs (n=4) and MMS (n=1) analyzed, some studies employed longer intervention durations to observe behavioral maintenance; however, these accounted for a relatively small proportion of the evidence base. Subgroup analysis demonstrated a progressive increase in the proportion of studies classified as “effective” with increasing intervention duration (see Table 3): 2 out of 4 (50.0%) for ultra-short-term (<1 week), 10 out of 16 (63%) for short-term (>1 and <8 weeks), 12 out of 18 (67%) for medium-term (8-16 weeks), and 3 out of 4 (75%) for long-term (>16 weeks). Notably, medium-duration interventions (8-16 weeks) not only represented the largest proportion of the existing evidence but also demonstrated both a relatively high “effective” rate (12/18, 67%) and a low “ineffective” rate (1/18, 6%). These findings indicate that current DHI research remains skewed toward short- and medium-term interventions, with the 8-16-week category standing out in terms of evidence volume and the apparent stability of intervention effects.

**Table 3:** Subgroup analysis of intervention duration.

Duration group (weeks)	Number, n	Effective (yes), n (%)	Limited effect, n (%)	Not effective (no), n (%)
Ultrashort (≤1)	4	2 (50.0)	2 (50.0)	0 (0)
Short (>1 and <8)	16	10 (63)	4 (25)	2 (13)
Medium (8-16)	18	12 (67)	5 (28)	1 (6)
Long (>16)	4	3 (75)	0 (0)	1 (25)
Subtotal (analyzed)	42	27 (64)	11 (26)	4 (10)
Excluded: not reported	4	N/A^a^	N/A	N/A

^a^N/A: not applicable.

#### Intervention Outcomes

As a result of substantial heterogeneity among the included studies with respect to outcome measurement instruments, outcome definitions, and assessment time points, it was not feasible to define a unified primary outcome or to conduct a statistically valid meta-analysis. Accordingly, this review adopted a descriptive synthesis framework to summarize and integrate the relevant outcomes. The outcome metrics in the included studies were classified into 2 main categories. The primary outcomes focused on lifestyle behaviors, including physical activity (eg, activity level, step count, and activity intensity), sedentary behaviors (eg, total sedentary time and frequency of breaks from sitting or resting), diet (eg, dietary quality; intake of fruits, vegetables, and sugar-sweetened beverages; energy intake; and nutritional knowledge), and sleep (eg, sleep quality, duration, efficiency, and severity of insomnia). These indicators directly reflect changes in core health behaviors resulting from the intervention and serve as a key basis for evaluating its effectiveness. Secondary outcomes, serving as supplementary indicators, were more diverse and encompassed physical health status and psychosocial dimensions, such as weight and body composition (eg, weight, BMI, waist circumference, and body fat percentage), physical fitness indicators (eg, flexibility, muscle strength, and cardiorespiratory fitness), cardiometabolic indicators (eg, blood pressure, blood glucose, and blood lipid profiles), and psychological and self-perception measures (eg, self-efficacy, body image, and life satisfaction). Overall, current studies remain primarily focused on primary outcomes, while secondary outcomes have expanded but continue to exhibit limited coverage.

#### Intervention Effectiveness

Based on the reported effect measure types, effect estimates, confidence levels (%), and CIs across the included studies, together with a comprehensive assessment of the authors’ conclusions (see Multimedia Appendix 3), the results indicated that 31 (67%) studies demonstrated evidence of intervention effectiveness, suggesting that DHIs are generally associated with positive outcomes in improving lifestyle behaviors among college students. Four studies reported no statistically significant effects, with limitations primarily attributed to small sample sizes or short intervention durations. The remaining 11 studies demonstrated limited effectiveness, with improvements observed only in selected secondary outcomes or during short-term follow-up periods.

Based on a comprehensive assessment of each behavioral domain using the Grading of Recommendations Assessment, Development and Evaluation (GRADE) framework, the certainty of evidence for the physical activity and diet domains was rated as “moderate,” whereas the evidence for the sedentary behavior and sleep domains was rated as “low.” With respect to evidence credibility, this review indicates a moderate level of confidence in the overall estimate that DHIs are effective in improving lifestyle behaviors among college students. The certainty of evidence in some domains was downgraded due to methodological limitations in the existing primary studies, including small sample sizes, challenges in implementing blinding, and inconsistencies in outcome assessment tools. Nevertheless, these GRADE assessments provide an accurate reflection of the current state of the evidence and its overall strength for DHIs among college students, thereby offering valuable guidance for interpretation and future research.

## Discussion

### Principal Findings

#### Discussion on Current Research Status

In terms of temporal trends, research on DHIs targeting college students’ lifestyle behaviors has gradually emerged since 2014, expanded rapidly after 2016, and reached a peak in the past 5 years [6]. This trend has been driven primarily by 4 categories of factors. First, technological advances have laid a solid foundation for DHIs, with the proliferation of smartphones, wearable devices, and app ecosystems significantly enhancing their accessibility and operability [67]. Second, conceptual advancements have accelerated theoretical and methodological innovations in DHIs, underscoring their distinctive advantages in facilitating behavioral improvement [68]. Third, demand has increased substantially, particularly during the COVID-19 pandemic, with DHIs gaining broad recognition as viable alternatives when traditional approaches were constrained [14]. Fourth, resource investment has continued to expand, with funding, supportive policy frameworks, and interdisciplinary collaboration creating a favorable environment for research. Overall, future research is expected to shift from assessing short-term feasibility to evaluating long-term effectiveness, scalability, and the capacity to accommodate personalization [29,30].

In terms of spatial distribution, research on DHIs is predominantly concentrated in high-income countries, particularly in the United States and Australia. This concentration is primarily driven by a combination of technological infrastructure, research resources, and supportive policy environments. On the one hand, North America and Oceania initiated mHealth development relatively early, benefiting from substantial technological and financial advantages [31]. On the other hand, colleges in these regions generally possess mature health promotion systems and well-established ethical review mechanisms, facilitating the implementation of intervention trials. In addition, higher levels of health awareness and greater digital acceptance in Western cultures further contribute to this pattern. However, the generalizability of these findings may be limited when extrapolated to low- and middle-income countries. For example, resource-constrained settings may encounter infrastructural and hardware-related barriers, such as uneven network coverage and low rates of digital device ownership. Furthermore, substantial cross-cultural variations exist in perceptions of privacy, the role of family involvement, and prevailing health communication practices. In the future, cross-cultural validation and localized adaptation of DHIs should be strengthened [69,70], particularly in resource-constrained settings. Moreover, the development of low-cost, low-threshold DHI models should be explored to advance global health equity [11,71].

In terms of population structure, current research on DHIs has predominantly focused on generally healthy college students, a focus attributable to this group’s modifiable health behaviors and susceptibility to environmental influences. However, some studies have extended to special populations, including college students with overweight or obesity, individuals at risk for eating disorders [15,32], and students experiencing sleep disorders or psychological stress [33,34]. This differentiation strategy is partly motivated by the fact that special populations face higher health risks, thereby increasing the potential benefits and clinical value of interventions [72,73]. It also aligns with the need for precision interventions and stratified management. However, existing research has not yet sufficiently examined variations in engagement levels among students from diverse sociodemographic backgrounds. Limited attention to factors such as socioeconomic status and access to digital devices may result in disproportionate benefits for students with greater financial or digital resources, while those experiencing economic constraints or limited device access may be marginalized in the intervention process. Accordingly, future research is likely to advance along 2 complementary directions: first, continuing large-scale studies targeting general undergraduate populations to assess the generalizability of interventions; and second, strengthening targeted interventions for high-risk groups while prioritizing the reduction of participation barriers among students from diverse backgrounds [74]. Such efforts may drive the development of DHIs toward greater refinement, equity, and personalization.

#### Discussion on Intervention Implementation

In terms of intervention objectives, physical activity and diet are the 2 lifestyle behavior categories receiving the most research attention [35], whereas sedentary behavior and sleep are relatively underrepresented. Both single-behavior and multibehavior combined interventions coexist. This pattern is primarily influenced by several factors. First, physical activity and diet are directly associated with weight management, energy balance, and metabolic health—core variables that affect college students’ physical fitness and chronic disease risk [36]. Related measures (eg, step count, energy intake) are more easily quantifiable and standardized, making them more likely targets for intervention. By contrast, sedentary behavior and sleep, despite their recognized importance [37], pose technical and operational challenges for DHIs, including measurement complexity and delayed feedback on intervention effects [38], contributing to a relative paucity of research [39,40]. Current DHIs demonstrate limited effectiveness in reducing sedentary time among college students [75], whereas sleep interventions, although promising, remain understudied and predominantly focus on insomnia relief [18,76]. Second, intervention strategies reflect researchers’ assessment of behavioral variability: physical activity and diet exhibit a wide window for controllability and modification, whereas sedentary behavior typically occurs in academic or leisure contexts, complicating immediate adjustment via a single technique. The prevalence of single-behavior interventions is attributed to their suitability for early exploratory phases, allowing easier control of variables and validation of intervention effects. Conversely, the increase in multibehavior interventions reflects the aggregation of lifestyle risks among college students, which complicates achieving sufficient health benefits through changes in a single behavior. Notably, the combination of physical activity and diet is the most frequent, reflecting the necessity for integrated interventions targeting weight management and energy metabolism [41]. Overall, future research is expected to increasingly adopt multibehavior approaches, integrating behavioral science theories and technological tools to develop synergistic interventions that address the complexity of lifestyle risks.

In terms of intervention modalities, an evolutionary trend is evident, progressing from single to multiple formats and from low to high levels of interaction, driven by the combined forces of technological advancement, user demand, and intervention science. In early studies, SMS text messages and emails were the predominant forms of DHIs [42], owing to their low technological threshold, ease of deployment, and minimal cost, which made them suitable for rapid implementation in resource-limited contexts [43,44]. However, these approaches primarily involved 1-way information delivery, lacked personalization and real-time interaction, and were insufficient in maintaining user engagement. With the widespread adoption of smartphones and the maturation of the app ecosystem, mobile apps have gradually become the mainstream form of DHIs. These apps are highly integrated and interactive, capable of incorporating multiple functions such as goal-setting, feedback, reminders, and data tracking [13], aligning with college students’ high-frequency mobile usage habits and significantly enhancing the intervention experience and engagement [45]. Web platforms retain advantages in scalability but are somewhat less user-friendly and less effective in delivering push notifications compared with apps [46,47]. In addition, the integration of social media and wearable devices increases the interactivity and contextual adaptability of DHIs [48], further enhancing behavioral monitoring and the provision of immediate feedback [49-51]. Future trends are expected to emphasize technological convergence and intelligent development. On the one hand, combinations of multiple formats (eg, apps, social platforms, and gamification) will become increasingly prevalent to address the multidimensional needs of behavioral interventions [52]. On the other hand, personalized interventions leveraging artificial intelligence, virtual coaching, and immersive experiences (eg, augmented reality/virtual reality) are anticipated to emerge as key research directions [17,53], shifting DHIs from being information-driven to experience-driven and ultimately facilitating sustained behavior change.

In terms of intervention functions, current DHIs are predominantly characterized by education, guidance, monitoring, and prompting components [54], indicating that these interventions primarily emphasize information delivery and basic behavior management. This design approach is partly driven by the substantial demand for health knowledge and skills among college students, with education and guidance functions facilitating improvements in cognition and self-efficacy. Concurrently, monitoring and prompting functions leverage technology to enable data recording and behavioral reinforcement, thereby promoting the initiation of target behaviors in the short term [55]. However, high-engagement features such as feedback, interaction, and gamification-based incentives remain underutilized [56,57], suggesting that DHIs often lack deep personalization and social support components [58], which may be a critical factor limiting long-term user engagement and intervention effectiveness. In terms of future trends, the convergence of behavior change theories (eg, Capability-Opportunity-Motivation-Behavior [COM-B], behavioral economics) with intelligent algorithmic applications is expected to drive the evolution of DHI functionality toward greater personalization, interactivity, and emotional engagement [59]. For example, artificial intelligence–driven real-time feedback could enhance intervention adaptability, virtual communities could strengthen social support, and gamification mechanisms coupled with reward systems could foster intrinsic motivation. Such advancements are likely to not only increase intervention engagement but also substantially improve behavioral maintenance, fostering a gradual shift from information delivery–oriented DHIs to approaches that place greater emphasis on user experience and social interaction.

#### Discussion on Intervention Effectiveness

Overall, 31 of 46 (67%) studies reported effective outcomes (yes), indicating the high feasibility and considerable potential of DHIs in improving the lifestyle behaviors of college students [60-62,77]. However, a subset of studies yielded insignificant (no) or limited (limited) effects, which can be examined from several analytical dimensions. First, insufficient refinement and lack of theoretical underpinning in intervention design represent key factors constraining effectiveness. In several cases, interventions lacked explicit theoretical frameworks for behavior change, relying predominantly on information delivery. Such approaches often failed to sufficiently stimulate participant motivation or reinforce behavior maintenance, leading to short-term gains that were difficult to sustain [26]. Second, existing intervention studies generally lack robust validation of long-term effects. Most studies are limited to durations of 8-16 weeks and include insufficient follow-up, which constrains the ability to verify the sustainability and stability of behavioral changes [63]. As a result, the long-term value and durability of DHIs remain difficult to assess adequately. Third, intervention effectiveness appears to be strongly influenced by participant adherence. Analyses of engagement-related metrics indicate that higher levels of user engagement, compliance, and intervention consistency are generally associated with more favorable behavioral and clinical outcomes. By contrast, studies characterized by high dropout rates often rely predominantly on 1-way information delivery, with limited opportunities for feedback and interaction. Fourth, the type of target behavior and associated measurement challenges also contribute to these outcomes. Compared with physical activity, the intervention effects on diet, sedentary behavior, and sleep were more vulnerable to external environmental influences (eg, academic workload, dietary contexts), and measurement tools relied predominantly on self-reporting, thereby increasing bias and uncertainty. Taken together, variations in intervention design, technological application, and behavioral characteristics collectively contribute to the substantial heterogeneity observed in intervention outcomes [64].

To gain a deeper understanding of variations in intervention effectiveness, the COM-B framework can be applied as a systematic analytical tool [78]. (1) Within the “Capability” dimension, most interventions primarily enhanced college students’ health-related knowledge through educational content and guidance materials. Examples included the provision of diet guidelines, exercise plans, and sleep regulation strategies designed to increase participants’ awareness of the importance of healthy behaviors. However, these improvements often remained at the cognitive level, with limited emphasis on the development of practical behavioral skills. Specific components, such as diet substitution options, situational coping strategies, or flexible exercise planning, were frequently absent. In addition, some studies did not provide adequate support for data interpretation, which limited participants’ ability to translate behavioral monitoring data into actionable steps [79]. (2) Within the “Opportunity” dimension, DHIs generally rely on virtual platforms to create enabling behavioral conditions, such as goal tracking, reminder functions, and online resource sharing, which may theoretically reduce psychological barriers to behavior enactment. However, the structuring of opportunities within real-world contexts remains insufficiently optimized. Some interventions do not adequately account for the distinctive time pressures and contextual constraints experienced by college students on campus. For example, strategies aimed at reducing sedentary behavior often remain limited to generic standing reminders, without adaptation to classroom environments or common study spaces, thereby constraining opportunities for sustained behavior change. Furthermore, although some interventions attempt to incorporate social support mechanisms (eg, community interactions or peer challenges), the depth and quality of participant engagement are generally limited. These interactions frequently involve 1-way information transmission, with limited capacity to foster emotional connection or effective behavioral modeling. (3) Within the “Motivation” dimension, existing interventions primarily emphasize the stimulation of extrinsic motivation through short-term incentives, such as point-based rewards and task completion reminders. While such strategies may promote initial engagement, they generally lack mechanisms for the sustained cultivation of intrinsic motivation. Specifically, many interventions have not effectively supported college students in developing a sense of self-worth derived from continued engagement in healthy behaviors. In addition, strategies aimed at enhancing positive emotional experiences are rarely incorporated. For example, gamification designs often remain confined to superficial point-based systems, with limited capacity to stimulate participants’ sense of exploration, mastery, or accomplishment. Additionally, insufficient personalization of feedback appears to substantially constrain the maintenance of motivation over time. Participants often receive generic informational messages rather than timely, individualized feedback closely aligned with their actual behavioral performance.

In summary, current DHIs predominantly adopt a “technology-driven” or “utility-oriented” design logic, with a primary emphasis on functional implementation and surface-level engagement metrics. Because of the limited integration of behavior change theory, such interventions tend to exhibit constrained effectiveness in sustaining long-term outcomes. By contrast, theory-driven interventions—such as those grounded in the COM-B framework—extend beyond short-term behavior initiation, emphasizing the synergistic development and dynamic support of capability, opportunity, and motivation. Through structured and phased behavioral support strategies, such interventions may facilitate the establishment of enduring foundations for sustained change across cognitive, skill-based, environmental, and emotional dimensions [10]. As a result, long-term behavior maintenance may become more attainable [65]. Future research should further position behavior change theory as a central guiding principle in intervention design, moving beyond the view of technology as a standalone tool and instead embedding it organically within support systems centered on behavior change mechanisms.

### Strengths and Limitations

This study is among the first English-language reviews to systematically integrate multiple forms of DHIs and multiple lifestyle behavior domains within a core population of college students, and it presents the following strengths. First, the study design strictly adheres to PRISMA 2020 and was preregistered on PROSPERO. The systematic search spanned 10 major international databases, ensuring the comprehensiveness and representativeness of the evidence base. Second, by focusing on college students as “digital natives,” this study systematically analyzes intervention characteristics across 4 health behavior domains—physical activity, sedentary behavior, diet, and sleep—thereby addressing limitations of prior reviews that emphasized a single behavior or tool. Third, drawing on the COM-B framework, this study examines the mechanisms of DHIs across the Capability, Opportunity, and Motivation dimensions and identifies key bottlenecks in intervention strategies—such as limited technological functionality, suboptimal ecological adaptability, and insufficient motivational activation—thereby providing both theoretical support and practical guidance for the future design and optimization of DHIs for college students.

Although this review endeavored to incorporate the existing literature as comprehensively as possible, several limitations remain. First, the geographical distribution of the included studies was uneven, with a heavy concentration in high-income countries—particularly North America and Australia—which constrains the global applicability of the findings; specifically, their generalizability to college students in low- and middle-income countries requires empirical verification. Second, many studies employed small samples, short intervention durations, and limited follow-up, and some lacked robust control groups or adequate randomization, thereby weakening the stability of effect estimates and the strength of causal inference. In addition, DHIs were often relatively homogeneous, with limited multidimensional interactivity and personalization; blinding procedures were difficult to implement; and risks of bias arose in adherence assessment and outcome measurement. Therefore, future research should strengthen sample representativeness, enhance intervention refinement, and improve methodological rigor to increase the external validity and practical utility of the findings.

### Implications and Recommendations

#### Recommendations for Policy and Practice

To fully realize the potential of DHIs while ensuring the sustainability and broad accessibility of intervention effects, systematic improvements in policy design and implementation pathways are required. First, college students should be explicitly incorporated into national and regional digital health strategies to facilitate a shift from traditional health education toward integrated digital platforms, and higher-education institutions should be encouraged to develop or adopt scientifically grounded, standardized tools with clearly articulated mechanisms of action. Second, localized development of intervention content and functionality should be supported, with attention to adaptability across behavioral domains, cultural contexts, and student needs, thereby advancing refined, human-centered design with respect to technological thresholds, data security, and personalized recommendations. Third, intervention practice should strengthen students’ active engagement and establish feedback-driven, behavior-reinforcing, and peer-support mechanisms to enhance sustained use and intrinsic motivation. In parallel, cross-departmental cooperation mechanisms should be established at the college level, and health interventions should be embedded within curricula, psychological support systems, and campus service resources to form a synergistic support network. Finally, at the policy level, ethical oversight and effectiveness evaluation of DHIs programs should be strengthened, and an evidence-based evaluation framework for DHIs should be established to ensure fairer, more adaptable, and more effective interventions for college students.

#### Recommendations for Future Research

This study indicates that current research on DHIs for college students remains constrained by unrepresentative samples, single-focus intervention content, and unclear technological mechanisms; future work should be refined and deepened in the following respects. First, geographical and cultural diversity should be expanded, prioritizing studies from low- and middle-income countries, varied higher-education institution types, and diverse social groups to enhance the external validity of the findings. Second, the design and evaluation of multibehavior-integrated interventions should be strengthened by moving beyond single-behavior paradigms and examining behavioral synergies and optimal combinations of intervention components. Third, higher-quality study designs—such as QRCTs, MMSs, and long-term follow-up—should be employed to strengthen causal inference and the sustainability of intervention effects. Fourth, theoretical development and empirical testing of intervention mechanisms should be strengthened by grounding analyses in behavior change theory to clarify how technology enhances Capability, Opportunity, and Motivation, and to advance DHIs from merely providing technical functions to creating a supportive ecosystem conducive to sustained behavior change. Finally, future studies should emphasize the assessment of intervention equity, systematically account for potential moderators such as gender, socioeconomic status, and psychological status, and identify subgroups with limited responsiveness, thereby providing a robust evidence base for constructing a more inclusive and adaptive DHI model for college students.

### Conclusions

This review addresses a gap in the literature by focusing specifically on college students, a group often overlooked in research that typically centers on broader adult populations. Unlike prior reviews that mainly examine a single lifestyle behavior, this study adopts a more holistic approach by integrating multiple behaviors and evaluating a range of DHIs with diverse modalities and functionalities. These findings provide valuable insights for refining future DHIs targeting college students and contribute to the development of more effective health promotion strategies in higher education. Although DHIs show potential for improving lifestyle behaviors, their long-term effectiveness remains uncertain. Current interventions face several limitations, including a narrow behavioral focus, basic technological functionality, and limited adaptability to diverse contexts, all of which may restrict long-term engagement and personalized responsiveness. Moreover, many interventions do not fully account for variations in resource access and individual behavior change pathways, potentially limiting their applicability and equity. Future research should prioritize integrating multiple behaviors, enhancing user engagement, improving contextual adaptability, and expanding technological accessibility. Long-term studies and equity-focused evaluations are essential for strengthening the evidence base and ensuring the sustainability and inclusivity of health behavior change among college students.

## Appendix

Multimedia Appendix 1 PRISMA (Preferred Reporting Items for Systematic Reviews and Meta-Analyses) checklist.

Multimedia Appendix 2 Search strategy used in this review.

Multimedia Appendix 3 Comprehensive data extraction table of included studies.

## Abbreviations

BCTTv1: Behavior Change Technique Taxonomy version 1
COM-B: Capability-Opportunity-Motivation-Behavior
DHI: digital health intervention
GRADE: Grading of Recommendations Assessment, Development and Evaluation
JBI: Joanna Briggs Institute
mHealth: mobile health
MMAT: Mixed Methods Appraisal Tool
MMS: mixed methods study
PICOS: Population-Intervention-Comparison-Outcome-Study Design
PRISMA: Preferred Reporting Items for Systematic Reviews and Meta-Analyses
PRISMA-S: Preferred Reporting Items for Systematic Reviews and Meta-Analyses—Search Extension
PROGRESS-Plus: Place of Residence, Race/Ethnicity, Occupation, Gender/Sex, Religion, Education, Socioeconomic Status, Plus Other Relevant Factors
PROSPERO: International Prospective Register of Systematic Reviews
QDS: quantitative descriptive study
QNRS: quantitative nonrandomized study
QR: qualitative research
QRCT: quantitative randomized controlled trial
RoB 2: Risk of Bias 2

## Data Availability

Data are provided within the manuscript or multimedia appendices; data/materials can be shared upon reasonable request to the corresponding author (ZHY).
