# Supplementary material for: Effect of Digital Health Interventions on College Students’ Lifestyle Behaviors: Systematic Review
Source: J Med Internet Res. 2026 Feb 4;28:e82192. doi: 10.2196/82192 (PMC12917487; doi:10.2196/82192)
Supplement: Multimedia Appendix 2 [file jmir_v28i1e82192_app2.docx]

**Appendix 2. Search Strategy Used in This Review**

| **Search Date** | | **Platform/Database** | | **Search strategy** | **Results** |
| --- | --- | --- | --- | --- | --- |
| **Initial search** | **Updated search** |  |  |  |  |
| August 5, 2025 | December 27, 2025 | Scopus | | (TITLE-ABS-KEY("digital health" OR "eHealth" OR "mHealth" OR "mobile health" OR "digital intervention" OR "health app"))  AND  (TITLE-ABS-KEY("college students" OR "university students" OR "undergraduate students"))  AND  (TITLE-ABS-KEY("lifestyle behavior" OR "health behavior" OR "physical activity" OR "exercise" OR "diet" OR "nutrition" OR "sleep" OR "sedentary behavior")) | 643 |
| August 5, 2025 | December 27, 2025 | Web of Science | | TS=("digital health" OR "eHealth" OR "mHealth" OR "mobile health" OR "digital intervention" OR "health app")  AND  TS=("college students" OR "university students" OR "undergraduate students")  AND  TS=("lifestyle behavior" OR "health behavior" OR "physical activity" OR "exercise" OR "diet" OR "nutrition" OR "sleep" OR "sedentary behavior") | 341 |
| August 5, 2025 | December 27, 2025 | PubMed | | ("digital health" OR "mHealth" OR "eHealth" OR "mobile health" OR "digital intervention" OR "health app")  AND  ("college students" OR "university students" OR "undergraduate students")  AND  ("lifestyle behavior" OR "health behavior" OR "physical activity" OR "exercise" OR "diet" OR "sleep" OR "sedentary behavior")  AND  ("intervention" OR "behavior change" OR "effectiveness" OR "impact")  AND  ("randomized controlled trial" OR "RCT" OR "experimental study" OR "intervention study")  NOT  ("review" OR "meta-analysis" OR "systematic review") | 76 |
| August 5, 2025 | December 27, 2025 | ProQuest Central | | ("digital health" OR "ehealth" OR "mhealth" OR "mobile health" OR "digital intervention" OR "health app")  AND  ("college students" OR "university students" OR "undergraduate students")  AND  ("lifestyle behavior" OR "health behavior" OR "physical activity" OR "exercise" OR "diet" OR "sleep" OR "sedentary behavior")  AND  ("intervention" OR "impact" OR "effectiveness" OR "behavior change")  AND  ("randomized controlled trial" OR "rct" OR "experimental design" OR "intervention study")  AND  NOT ("review" OR "meta-analysis" OR "systematic review") | 40 |
| August 5, 2025 | December 27, 2025 | EBSCOhost platform | MEDLINE | (("Digital Health"[Mesh] OR "Mobile Applications"[Mesh] OR "Telemedicine"[Mesh] OR "mHealth"[tw] OR "eHealth"[tw] OR "digital intervention"[tw] OR "health app"[tw]))  AND  (("Students"[Mesh] OR "college students"[tw] OR "university students"[tw] OR "undergraduate students"[tw]))  AND  (("Life Style"[Mesh] OR "Health Behavior"[Mesh] OR "Motor Activity"[Mesh] OR "Exercise"[Mesh] OR "Sleep"[Mesh] OR "Feeding Behavior"[Mesh] OR "sedentary behavior"[tw] OR "physical activity"[tw] OR "diet"[tw])) | 871 |
| August 5, 2025 | December 27, 2025 |  | PsycINFO | ("digital health" OR "ehealth" OR "mhealth" OR "mobile health" OR "digital intervention" OR "health app")  AND  ("college students" OR "university students" OR "undergraduate students")  AND  ("lifestyle behavior" OR "health behavior" OR "physical activity" OR "exercise" OR "diet" OR "nutrition" OR "sleep" OR "sedentary behavior") | 912 |
| August 5, 2025 | December 27, 2025 |  | SPORTDiscus | ("digital health" OR "mhealth" OR "ehealth" OR "mobile health" OR "digital intervention" OR "health app")  AND  ("college students" OR "university students" OR "undergraduate students")  AND  ("lifestyle behavior" OR "health behavior" OR "physical activity" OR "exercise" OR "diet" OR "sleep" OR "sedentary behavior") | 26 |
| August 5, 2025 | December 27, 2025 |  | APA PsycArticles | ("digital health" OR "mhealth" OR "ehealth" OR "mobile health" OR "digital intervention" OR "health app")  AND  ("college students" OR "university students" OR "undergraduate students")  AND  ("lifestyle behavior" OR "health behavior" OR "physical activity" OR "exercise" OR "sleep" OR "diet" OR "sedentary behavior")  AND  ("intervention" OR "behavior change" OR "effectiveness" OR "impact")  AND  ("randomized controlled trial" OR "rct" OR "experimental study" OR "intervention study")  NOT  ("review" OR "meta-analysis" OR "systematic review") | 3 |
| August 5, 2025 | December 27, 2025 |  | ERIC | ("digital health" OR "mhealth" OR "ehealth" OR "mobile health" OR "digital intervention" OR "health app")  AND  ("college students" OR "university students" OR "undergraduate students")  AND  ("lifestyle behavior" OR "health behavior" OR "physical activity" OR "exercise" OR "sleep" OR "diet" OR "sedentary behavior")  AND  ("intervention" OR "behavior change" OR "effectiveness" OR "impact")  AND  ("randomized controlled trial" OR "rct" OR "experimental study" OR "intervention study")  NOT  ("review" OR "meta-analysis" OR "systematic review") | 44 |
| August 5, 2025 | December 27, 2025 |  | Academic Search Premier | ("digital health" OR "ehealth" OR "mhealth" OR "mobile health" OR "digital intervention" OR "health app")  AND  ("college students" OR "university students" OR "undergraduate students")  AND  ("lifestyle behavior" OR "health behavior" OR "physical activity" OR "exercise" OR "sleep" OR "diet" OR "sedentary behavior")  AND  ("intervention" OR "effectiveness" OR "impact" OR "behavior change")  AND  ("randomized controlled trial" OR "rct" OR "intervention study" OR "experimental study")  NOT  ("review" OR "meta-analysis" OR "systematic review") | 42 |
